# Social Media Interventions for Nutrition Education Among Adolescents: Scoping Review

**DOI:** 10.2196/36132

**Published:** 2023-07-20

**Authors:** Yalinie Kulandaivelu, Jill Hamilton, Ananya Banerjee, Anatoliy Gruzd, Barkha Patel, Jennifer Stinson

**Affiliations:** 1 Child Health Evaluative Sciences The Hospital for Sick Children Toronto, ON Canada; 2 Centre for Healthy Active Kids The Hospital for Sick Children Toronto, ON Canada; 3 Dalla Lana School of Public Health University of Toronto Toronto, ON Canada; 4 Information Technology Management Toronto Metropolitan University Toronto, ON Canada

**Keywords:** adolescents, digital health intervention, food literacy, health literacy, nutrition, peer education, peer support, social media

## Abstract

**Background:**

Adolescence is a critical period for reinforcing healthy dietary behaviors and supporting the development of cooking skills. Social media may be an avenue for supporting these behaviors, as it is popular among adolescents and can improve access to nutrition education interventions. This study sought to understand the optimal implementation of effective social media–based nutrition education interventions to inform the implementation of future social media–based nutrition education interventions.

**Objective:**

A scoping review of the characteristics, feasibility, effectiveness, and factors influencing social media–based nutrition education interventions for adolescents was conducted.

**Methods:**

We searched MEDLINE, Embase, CINAHL, Web of Science, and PsycINFO databases using a predefined search strategy. Primary research articles were independently screened and included if they involved adolescent populations (10-18 years old) and delivered nutrition education through social media. The information on intervention characteristics, feasibility, effectiveness, and factors influencing social media–based nutrition education interventions was extracted.

**Results:**

A total of 28 publications out of 20,557 met the eligibility criteria. Twenty-five nutrition interventions were examined by 28 studies. Fourteen interventions used homegrown social media platforms, 8 used Facebook, and 2 used Instagram. Feasibility outcomes were infrequently reported, and the cost of intervention delivery was not reported. Engagement with interventions was variable; high engagement was not required to elicit significant improvements in dietary behaviors. Tailoring interventions, offering practical content, meaningful peer support, and involving families and communities facilitated successful interventions. Strategies to address engagement and technical issues were varied.

**Conclusions:**

Emerging evidence demonstrates that social media interventions for adolescent nutrition are acceptable and improve nutrition outcomes. Future interventions should strengthen peer support components and tailor delivery to specific populations. Further research should examine engagement, adherence, and the impact of interventions on behavioral and physical outcomes. This review is the first to examine the use of social media as the primary medium for nutrition education for adolescent populations. The analysis used in this review argues the importance of peer support in social media–based nutrition interventions and the need for user-centered design of the interventions.

## Introduction

Poor nutrition, such as the consumption of highly processed foods and infrequent consumption of vegetables, is directly linked to a multitude of preventable chronic illnesses, including ischaemic heart disease, diabetes, cancer, and poor mental health [1]. Dietary behaviors are influenced by a complex set of factors, including food security, social environments, attitudes and beliefs about food, and nutrition and food literacy [2-11]. Food and nutrition literacy are the skills and knowledge that allow individuals to prepare and eat good-tasting, healthy, and affordable foods on a daily basis and are linked to improved diet quality in adolescence [12,13]. Rates of preventable chronic illness will continue to increase, particularly in young people, if modifiable factors influencing nutrition are not addressed [14].

Cooking skills during adolescence also track into adulthood and are predictive of diet quality (eg, vegetable consumption) and confidence in cooking abilities in adulthood [15,16]. Behavioral patterns established during adolescence influence adolescents’ risk of developing chronic illnesses during adulthood and their current health. However, opportunities for learning cooking and food preparation skills have decreased in school and at home, and family meals have decreased due in part to parental precarious work, poor housing conditions, and the use of devices and media during mealtimes [17-20]. Thus, promoting food and nutrition literacy during adolescence is an important component of a multipronged chronic illness prevention strategy [21,22].

Interventions for food and nutrition literacy have positive impacts on adolescents’ nutrition knowledge, self-efficacy in cooking skills, and dietary behaviors [13,23-26]. Technology-based interventions, especially social media, are recommended to effectively engage adolescents, especially older adolescents [23,24]. Adolescents are especially sensitive to their social environments (eg, peers and social media) and use social media to socialize and learn new information, making social media an important avenue for health promotion [27,28]. Irrespective of income level, 95% of adolescents today use mobile phones, and upwards of 70%-80% of adolescents use social media (eg, Instagram or Snapchat) [28].

However, the effective design and implementation of social media interventions for nutrition in adolescents remain unclear [29]. One review of social media interventions for nutrition in adolescents and young adults found some positive impacts on BMI and dietary intake. However, the components of the interventions eliciting positive impacts, especially for adolescents from low socioeconomic status (SES) communities, remained unclear [29]. Adolescents from low SES communities are at the highest risk of poor nutrition due to food insecurity, uncertain or limited access to healthy foods, and lower food and nutrition literacy due to poor housing conditions and parental precarious working conditions [7,15-20,30-39]. To inform the development and evaluation of a social media–based food literacy intervention for adolescents from low SES settings, we sought to conduct a scoping review of the evidence on social media–based interventions for nutrition outcomes among adolescents. This review differs from previous reviews in several ways: we focused on adolescent populations as there are several implementation issues (eg, privacy and consent) and differences in food environments for this population (eg, living with parents or guardians); we used a broader search strategy and inclusion criteria, including earlier studies (2000 onward) and qualitative and quantitative studies, and we sought to identify any barriers and facilitators to the interventions and highlight factors that were specific to adolescents from low SES communities. Our research questions were:

1. What are the characteristics (eg, platform or frequency) of social media interventions used to address nutrition outcomes in adolescents?
2. What is the feasibility (eg, dropout rates or cost) of delivering interventions using social media for improving nutrition outcomes in adolescents?
3. What is the effectiveness of social media interventions in achieving positive changes in nutrition outcomes (eg, attitudes about nutrition, BMI, or dietary intake) among adolescents?
4. What factors influence the implementation and success of social media interventions for nutrition outcomes?

## Methods

We conducted a scoping review, as articulated by Arksey and O’Malley [40]. We chose a scoping review over a systematic review methodology due to our broader research questions and overarching aim to inform the development and evaluation of a social media–based food literacy intervention for adolescents from low SES settings [40].

### Literature Sources and Search Strategy

The search strategy was developed in collaboration with a medical library information specialist. It combined subject headings and text words relating to the main concepts using “AND” and “OR.” The search concepts were (1) social media, (2) children or adolescents, and (3) nutrition-related interventions. Searches of relevant electronic bibliographic databases (MEDLINE, Embase, CINAHL, PsycINFO, and Web of Science) for published work meeting the inclusion criteria were conducted, restricted to the years 2000 to April 7, 2022, with no language restrictions. We limited searches to the years 2000 and onward, corresponding with the emergence of social media for public use. We chose to include earlier studies with the earliest forms of social media because the essence of social media, which is peer-to-peer sharing and user-generated content, has remained consistent irrespective of the changes in platforms and technology over time. Reference lists of primary studies included in the review and of any relevant, previously published reviews were hand-searched. We initially reviewed the gray literature but decided not to include it to maintain the feasibility of the review. The gray literature we reviewed did not include evaluations of the interventions that were not already published in a peer-reviewed journal. A search strategy is available on request.

### Selection Criteria and Screening Process

We included publications in peer-reviewed journals meeting the following criteria: (1) population ages of 10-18 years old, as consistent with previous literature in this area and World Health Organization definitions of adolescence or described as adolescents; (2) social media as the primary intervention or a component of a complex intervention; (3) primary intervention provided nutrition education; and (4) conducted an evaluation of the social media intervention. All empirical study designs were included. Conference abstracts, guidelines, protocols, editorials or commentaries, dissertations, and reviews were excluded. Relevant reviews were examined for any potentially eligible studies, and dissertations were used to identify peer-reviewed publications. Social media was defined as electronic communications allowing for the creation of user-created communities and the sharing of information, personal messages, ideas, and audiovisual content [41]. Examples of social media include applications and websites such as Facebook, TikTok, and Instagram; forums (eg, Reddit); microblogging (eg, Twitter); and social bookmarking (eg, Pinterest) [42].

A calibration exercise was completed to ensure screening consistency and agreement; a random 10% subset of references was screened by 2 review authors (YK and BP), achieving an initial agreement of 97%. Any disagreements were resolved by discussion to achieve 100% agreement. Following the calibration exercise, the titles and abstracts of retrieved studies were screened independently by at least one review author (YK or BP) to identify studies that potentially met the selection criteria. The same exercise was completed again for full-text screening, achieving 95% agreement initially and 100% agreement following discussion. The screening was conducted using Covidence systematic review software [43].

### Data Extraction

Data extraction was conducted on Excel (Microsoft Corp) using a prepiloted form developed by the review authors to independently extract data [44]. Extracted information included: study context (eg, country and setting); intervention characteristics (eg, platform and content); population demographics (eg, ages and gender); study methodology (eg, study design); quantitative and qualitative study results; and study discussion and conclusions.

### Data Synthesis

We descriptively summarized study characteristics, intervention characteristics, and feasibility outcomes that were extracted from the included studies. Qualitative results from studies were analyzed using qualitative content analysis to identify and summarize factors influencing the implementation and success of the intervention. Data included in qualitative analyses included survey data, focus group discussions, individual interviews, and observation and field notes made by research staff during programs.

## Results

### Study Characteristics

A total of 20,557 unique references were retrieved through database searches and screened for inclusion based on the selection criteria. Of these, 28 studies of 25 distinct interventions met the inclusion criteria and were included in the review. See Multimedia Appendix 1 for the PRISMA (Preferred Reporting Items for Systematic Reviews and Meta-Analyses) flow diagram, including reasons for exclusion at the full-text screening stage. Fourteen studies were from the United States, 3 from Spain, 2 from Malaysia, 2 from Canada; and the remainder were from Brazil, Denmark, France, Indonesia, Portugal, and South Korea. Eleven studies were randomized controlled trials, 3 of which were cluster randomized controlled trials; 8 studies were pre-post designs, 2 of which were feasibility studies; 4 studies were quasi-experimental pre-post with control group designs; 2 studies were process evaluations; 2 studies were qualitative evaluations; and 1 study was a usability study. See Table S1 in Multimedia Appendix 2 for the characteristics of the included studies.

### Study Participant Characteristics

Total sample sizes ranged from 13 participants to 2001 participants. Four interventions were for females only; 11 studies had mostly female participants (60% or more); and 2 studies did not report the sex or gender of participants. Sixteen studies reported on race or ethnicity, 13 of which were from the United States, 1 from Malaysia, 1 from Canada, and 1 from Spain and Mexico. Of the 13 studies from the United States, 7 had mostly (50% or higher) Caucasian participants, 9 studies reported a proportion of African American participants (mean 22%; range 1.4%-46.2%), 7 studies reported a proportion of Latino or Hispanic participants (mean 20%; range 7.3%-43.5%), 1 study reported 16% of Asian participants, 1 study reported 4.9% American Indian or Alaska Native participants, 1 study targeted only Korean American participants, and 8 studies reported proportion of other or multiethnic categories (mean 17.6%; range 7.6%-46.7%). Four studies reported on the SES of participants using varying definitions and categorizations of SES, summarized in Table S1 in Multimedia Appendix 2. Eleven studies targeted adolescents who were considered overweight or obese. See Table S1 in Multimedia Appendix 2 for characteristics of included studies.

### Social Media Interventions

Intervention durations were between 6 weeks and 2 years with the most common length being 12-16 weeks (9/22, 41%; 3 studies did not report length). Most interventions used homegrown websites with social networking functions (10/25, 40%), 4 (16%) interventions used homegrown mobile phone apps, 8 (32%) used Facebook, 2 (8%) used Instagram, and 1 (4%) used an unspecified social networking service. Homegrown platforms included discussion forums, chat functions, resource centers, games, and education modules. Thirteen studies involved additional intervention components (17/25, 68%), including attendance at a weight management clinic, intermittent face-to-face group meetings or meetings with a health professional, text message reminders, weekly newsletters, weekly quizzes, web-based rewards for healthy goal setting, informational websites, changes to the school curriculum, a food diary, exergaming, and a new food labeling system at the school cafeteria. In addition to nutrition education, 16 interventions addressed physical activity and exercise; 7 interventions addressed weight management, weight loss, and obesity; 5 interventions addressed eating disorders, body image, or related symptoms and behaviors; and 2 interventions addressed stress management. Nine interventions (9/25, 36%) involved parents, with parent-specific websites or newsletters, or providing intervention access to parents. Ten interventions (10/25, 40%) reported how they were developed, 8 used feedback from or the involvement of adolescents to design the intervention, and 2 used an iterative user-centered design process. Four interventions were designed by educators or health care providers. No interventions reported including a cooking skills component to the intervention. See Table S2 in Multimedia Appendix 3 for a summary of social media intervention characteristics.

### Feasibility

Most studies did not report recruitment rates (17/25, 26%), while 3 (16%) recruited participants in a short period of time (eg, 1-3 weeks) at the start of the intervention. Twelve studies reported dropout rates, ranging from no dropouts to 68% dropout; however, most (7/12) were between 0% and 22%. Eight studies (8/24, 33%) reported some program costs: 5 gave participants gift certificates or compensation (US $20 to US $50) for outcome measures or study completion; 2 used prize contests to motivate increased participation; 2 provided participants with equipment (eg, iPhone and digital food scale) to participate in the intervention; 1 allowed participants to “cash in” points from adhering to the intervention to redeem at local merchants; and 1 provided a US $25 monthly incentive to assist participants with mobile data costs [45]. See Table S3 in Multimedia Appendix 4 for a summary of feasibility outcomes.

### Factors Influencing Implementation and Success of Interventions

### Overview

Factors influencing the implementation and success of interventions were categorized into the following themes: role of families and communities, tailoring interventions for the target population, engagement, technical and logistical issues, and peer support. See Table S4 in Multimedia Appendix 5 for a complete summary of categories.

#### Role of Families and Communities

Studies reported that parents’ inclusion in programs elicited positive impacts on participants, irrespective of their level of involvement, and that positive behavior changes among participants diffused to their families and peers [46-53]. When considering parents’ inclusion in programs, studies suggested that parental digital literacy needed to be considered as well as whether their involvement would detract from the independence adolescents would be attempting to achieve during this period of development [47,53,54]. Conversely, the positive impact of including families in interventions needed to be balanced with intervention content that addressed interpersonal barriers to healthy eating, such as peer and family pressure, lack of parental support for healthy eating, lack of control over food at home and in social settings, and tensions with cultural foods and healthier food choices [52,55,56]. Designing interventions in concert with community partners aided in the planning and delivery of the intervention, identifying improvements, and ensuring intervention sustainability [48,57-62].

#### Tailoring for Target Population

Studies recommended selecting social media platforms according to the target population, as differences in usage of platforms may exist between genders, SES levels, age groups, and countries [47,48,52,54,55,63]. Adolescents preferred content presented in actionable terms; frequent opportunities for peer interaction; examples of good-tasting healthy food; minimal, low-cost ingredient simple recipes; and culturally relevant content [48,52,55,59,61,64,65]. Studies found that content should be reviewed ahead of delivery to avoid stigmatizing messaging and encourage healthy lifestyles [52,63,64,66-69]. Finally, including adolescents’ feedback at all stages of the research was effective in ensuring the relevance of interventions to youths [47,51,56,59,62,64].

#### Engagement

Participants were more motivated to engage with interventions when they were involved in existing peer networks and peer support and used social media platforms that they already use [48,57,59,63,64,68-70]. Strategies, such as notifications and message prompts; adjunct in-person meetings; peer leaders, educators, and mentors; and greater intervention guidance, facilitated engagement [45,48,57,58,64,67-70]. Low or passive engagement may be enough to elicit preliminary improvements in dietary behaviors [54,58]. However, for future interventions, dynamic and responsive social media environments similar to media that adolescents already engage with would be essential to maintain adolescents’ engagement in the intervention and nutrition education more broadly [48,67,71].

#### Technical and Logistical Issues

To ensure the success of interventions, studies recommended using platforms that participants already use, adapting to changes in social media, testing social media functionalities regularly, counseling participants on privacy and safety on the internet, creating privacy and safety contingency plans, and ensuring access to devices and the internet [45,48,52,59,62,65,70,72].

#### Peer Support

Nutrition education delivered through social media needed to involve meaningful peer support to facilitate sustainable changes in health behaviors [45,48,52,61,63,67-69]. Effective peer support was drawn from participants’ existing social networks, from individuals whose opinions and judgments they valued, and from those who were from similar backgrounds [45,48,52,61,63,67-69]. Several mechanisms of influence explain the role of peer support in health behavior change using social media [48,49,52,68,69]. Thus, studies reported some uncertainty regarding how interventions should be designed to facilitate health behavior change [48,49,52,68,69].

## Discussion

### Summary

We sought to review the literature on social media interventions for nutrition in adolescent populations. Twenty-eight studies met our eligibility criteria and were included in the synthesis. The interventions were highly varied, although generally, they used similar social media platforms (eg, Facebook, homegrown websites, or apps) and duration (12-16 weeks). The feasibility of interventions could not be adequately assessed due to a lack of reporting and varied dropout rates, engagement, and reported costs. Of the studies examining the effectiveness of interventions, improvements in BMI and dietary behaviors typically occurred between 6 and 9 months of follow-up. Finally, factors influencing interventions suggested that effective interventions must be tailored to populations, offer peer support, and be dynamic and responsive, similar to existing social media environments.

### Principal Results

Social media interventions varied in the platform, length, theoretical foundation, format, and content, similar to previous review findings [73,74]. Trends, such as the use of Facebook and websites, reflect the dominant social media platforms from 2007 to 2014 when the top social media platform used by adolescents was Facebook [75]. Yet, the trend in the use of homegrown apps and websites continued into later studies included in this review. This may be due in part to concerns regarding the safety of adolescents when using popular social media, which remains a challenge in delivering these interventions. Today, adolescents report using platforms such as Instagram and Snapchat exclusively on mobile phones [28]. Reviews of social media interventions for child health, HIV treatment and prevention, and smoking, and studies in this review suggest that the best practice is to use existing social media platforms that adolescents already use to increase ease of use and accessibility and reduce costs [76-79]. New platforms may be adopted according to current trends and the target population’s usage [76-79]. However, irrespective of technology and platform, studies reported challenges in maintaining adequate peer-to-peer sharing and support. Many studies reported using interpersonal-level theories to inform the design of interventions since a key feature of social media is peer-to-peer sharing. However, few interventions centered on using peer support to achieve behavior change, and the peer educators or mentors had a limited role in the few interventions where they were involved.

There was limited information on feasibility in the included studies. Dropout rates and engagement varied widely when reported, and recruitment rates and cost information were seldom reported. The promise of social media–based health interventions lies in their assumed ability to reach broader geographic regions, increase accessibility, be cost-effective, and effectively engage adolescents; however, no studies have examined the extent to which this occurs. Many existing nutrition education programs are delivered by dieticians or other licensed health professionals or by teachers in schools, have limits on the number of participants they may reach, and vary in terms of their ability to provide tailored content to adolescents, reach broader geographic regions, effectively engage adolescents, and achieve improvements in dietary behaviors [13,23-26]. A clearer understanding of the costs and benefits of the different types of programs can aid decision makers in determining appropriate interventions and sustainability.

The data on the effectiveness of interventions was variable. Some studies demonstrated effects on BMI and dietary behaviors up to a 6- to 9-month follow-up. Similar improvements in dietary behaviors were found in a review of social media interventions for diet and exercise [80]. However, our ability to conclude effectiveness is limited due to the heterogeneity of social media interventions and the presence of multicomponent interventions. While these findings suggest some positive impact of social media interventions on nutrition outcomes in adolescent populations, without an appropriate understanding of dose-response, it is difficult to generalize the findings of these studies to other interventions or populations. Future definitive trials focused on examining effectiveness and dose-response will be needed to better understand these interventions.

We grouped factors influencing interventions into several categories, including the role of families and communities, tailoring for population characteristics, engagement with the intervention, technical and logistical issues, and peer support.

A few studies examined the role of parents in the intervention and suggested that they facilitated engagement with the intervention. Studies that involved parents found that they were facilitators regardless of how involved they were [48-51,81]. Even the act of parents providing consent for adolescents to participate in the intervention had an effect on improving participation [48]. These findings suggest significant involvement among parents may not be an essential component of programs for adolescents and can reduce some of the challenges when targeting low-income or ethnic minority youths, where parental time constraints, language, digital literacy, and other barriers may prevent parents from being involved in the intervention.

Several studies emphasized the role of involving communities in ensuring engagement, sustainability, and success of the intervention. Evidence from a range of health promotion interventions suggests that community-based interventions are most successful in improving nutrition [77,82]. Community partners can continue to deliver successful programs after studies end, streamline recruitment, and increase the acceptability of the program as participants are more likely to participate in a program delivered by familiar people and organizations.

Studies unanimously recommended more tailored approaches to program delivery. However, there was limited information regarding the tailoring of interventions to low SES and ethnic minority populations. Many US-based studies mainly targeted White participants, and others did not report the race or ethnicity of participants, similar to the findings of the systematic review of social media for diet and exercise by Williams et al [80]. However, studies such as Januraga et al [52] reported that interventions need further modifications to resolve participants’ tensions between family and culture-centered values, their desire to make healthy changes to their diets, and the interpersonal barriers that adolescents face in advocating for their healthier choices to parents and peer circles. A US study that targeted Korean-American adolescents also reported a need for more culturally tailored information [56]. Evidence on health behavior change in ethnic minority populations suggests that culturally tailored and facilitated interventions are more likely to achieve improvements in health outcomes [83]. Social media interventions have an opportunity to address these gaps, as official and national dietary guidelines may not address the breadth of diverse populations to the same extent as social media [84]. Among studies designed with youths’ involvement, none reported the extent to which youths’ preferences for nutrition education content were included. Nutrition education in the interventions was developed based on a variety of sources. Due to the scope of this review, we were unable to contact the authors of studies for further information regarding the nutrition curriculum used for each intervention and examine whether it was evidence-based.

Many studies targeted female participants and had higher female participation than male participation. The focus on adolescent females may be because several studies had the concurrent aim of eating disorder prevention, which affects girls and women at higher rates [85]. However, poor dietary behaviors and obesity affect males at higher rates than females, and males are less likely to engage with health services or be targeted by programs or services than females [86-88]. Furthermore, no studies in this review addressed adolescents who identify along the continuum of gender identity or sexual orientation, despite these adolescents being at greater risk of poor dietary behaviors, obesity, and disordered eating [89].

Engagement with interventions was often reported as a challenge, and several strategies were suggested for improving engagement. Challenges with engagement are frequently reported in other social media–based health interventions, and strategies, such as end user involvement in intervention design and financial incentives for participation, are suggested as potential ways to improve engagement [80]. However, despite involving end users in intervention design and financial incentives, studies in this review still found difficulties in engagement. Instead, feedback from adolescents suggested that leveraging existing friendships and social networks would increase engagement. Furthermore, most interventions in this review were static, with intervention content developed and then delivered with no further changes. Interventions are often “competing” with social media and adolescents consume on a regular basis that involve real-time responsiveness to participant engagement and feedback and tailoring content as social media users interact with it. Social media–based interventions for HIV treatment and prevention that involve dynamic and responsive content and peer-to-peer interaction have found fewer challenges with engagement [76].

Where peer support was active and engaged, participants reported positive experiences and a preference for more opportunities for peer support, a preference that is in line with previous reviews of social media for adolescent health [77]. Reviews of social media interventions for HIV treatment and prevention find that those that involved significant peer or social support components (eg, mentors or the primary aim of peer-to-peer communication) had high participant satisfaction with peer and social support and were linked to higher testing rates [76,90]. The role of peer mentors or educators was minimal in most interventions in our review, with few offering formal training to peer mentors. However, should future interventions incorporate peer mentors or leaders, formal training may ensure the effectiveness of mentor support by providing mentors with appropriate skills and strategies to deliver interventions [91].

Several studies discussed the role of technical issues and the need for them to be solved in real time and addressed ahead of or during the intervention in usability testing. A few studies discussed issues related to privacy and consent for studies. As many studies used homegrown websites and applications, the study teams were able to have greater control over intervention privacy. Studies that used Facebook asked adolescents to create new accounts for the study itself or used private, anonymized pages or groups. This issue requires further attention to ensure the successful implementation of social media–based nutrition education for adolescents without increasing barriers to participation [74].

This review highlighted several potential areas for future research on social media interventions for adolescent nutrition to explore. First, given that the aim of social media is to promote peer-to-peer sharing and communication, interventions should ensure peer and social support remain central to the intervention and are measured as part of outcomes. Second, in the literature reviewed, few described tailoring to ethnic minority populations or targeting populations most vulnerable to poor nutrition. Addressing these issues will be important to reduce health inequities and ensure effectiveness among diverse populations. Many of the studies had a focus on adolescent females or generally had higher rates of adolescent females participating. The risk of overweight, obesity, and dietary behaviors tends to be higher in males versus females; thus, ensuring interventions are accessible and effective for different sexes and genders is crucial to reducing health disparities. Third, examining dose-response and essential components of interventions will improve estimates of improvements in outcomes and the successful implementation of programs. Identifying the appropriate length, frequency, and engagement with the intervention will improve program rollout and allow interventions to be replicated accordingly based on evaluations of dose-response of interventions. Last, none of the included studies examined the cost-effectiveness or cost of the intervention. Examining cost-effectiveness will be important for policy makers and decision makers.

### Strengths and Limitations

One of the strengths of this review was the inclusion of both qualitative and quantitative data, allowing them to be interpreted in context with one another. Examining both types allowed us to identify factors influencing social media interventions for nutrition among adolescents. The second was the comprehensive search strategy we developed in collaboration with a medical library information specialist. The search strategy ensured we addressed a broad range of social media and varieties of interventions addressing nutrition in adolescent populations and addressed several gaps in previous reviews. Third, our focus on adolescents allowed us to examine specific issues related to consent, privacy, and safety, as well as the role of parents and communities. Previous reviews of social media interventions for health have combined adolescents with young adult populations [29,42]. Finally, our focus on the peer-to-peer sharing aspects of social media allowed us to gain an understanding of the unique role of social media in nutrition and health promotion for adolescents.

The results of this scoping review must be interpreted in context with its limitations. Due to resource constraints, the authors of included studies were not contacted for missing or unpublished data which may have yielded potential information to address our research questions. This review did not examine in depth the nutrition education provided in the interventions as it was beyond the scope of the review; however, summaries of the nutrition education are provided in Table S2 in Multimedia Appendix 3. Studies came from different fields interested in nutrition education interventions, such as eating disorder prevention, weight management, and healthy eating promotion. Thus, the outcomes measured in certain studies are less applicable to other fields, and intervention design, development, and content focus differ depending on the health concern.

### Conclusions

Accessible and cost-effective health promotion targeting adolescents’ nutrition is an important component of a multipronged strategy for the prevention of chronic illnesses. Social media interventions are essential components of effective health promotion, as social media are ubiquitous among adolescents and afford the ability to provide education and psychosocial support in developing healthy eating. The results of this review demonstrate that social media interventions for adolescents’ nutrition are acceptable and demonstrate promising impacts on dietary behaviors. Further research is required to understand the dose-response of interventions, the role of parents in interventions, the design of programs tailored to ethnically diverse adolescents and adolescent males, the role of peer leaders and peer support in programs, the impact on health-related outcomes, and cost-effectiveness.

## Appendix

Multimedia Appendix 1 Prisma flow chart.

Multimedia Appendix 2 Table S1. Characteristics of included studies.

Multimedia Appendix 3 Table S2. Summary of social media intervention characteristics.

Multimedia Appendix 4 Table S3. Feasibility outcomes.

Multimedia Appendix 5 Table S4. Factors influencing implementation and success of interventions.

## Abbreviations

PRISMA: Preferred Reporting Items for Systematic Reviews and Meta-Analyses
SES: socioeconomic status
