# Supplementary material for: Social Media Interventions for Nutrition Education Among Adolescents: Scoping Review
Source: JMIR Pediatr Parent. 2023 Jul 20;6:e36132. doi: 10.2196/36132 (PMC10401194; doi:10.2196/36132)
Supplement: Multimedia Appendix 1 [file pediatrics_v6i1e36132_app1.docx]

Records identified through database searching
**(n = 21828)**

Duplicates removed
(n =1281)

Additional records identified through other sources
**(n = 10)**

**Identification**

Records after duplicates removed
**(n = 20557)**

**Screening**

Records excluded
(n = 20358)

Records screened
**(n = 20557)**

**Eligibility**

Full-text articles excluded, with reasons
(n = 171)

**86** No social media

**64** Adult population

**14** Not nutrition education

**3** No evaluation

**3** Wrong publication type

**1** Participant ages not reported

Full-text articles assessed for eligibility
**(n = 199)**

**Included**

Studies included in scoping review
**(n = 28)**

**Figure 1. PRISMA Flow Diagram.** Details the flow of information through the different phases of the review; maps out the number of records identified, included and excluded, and the reasons for their exclusion.
