# Supplementary material for: Social Media Interventions for Nutrition Education Among Adolescents: Scoping Review
Source: JMIR Pediatr Parent. 2023 Jul 20;6:e36132. doi: 10.2196/36132 (PMC10401194; doi:10.2196/36132)
Supplement: Multimedia Appendix 2 [file pediatrics_v6i1e36132_app2.docx]

**Table S1. Characteristics of included studies.**

|  | **Author** | **Country** | **Study Design** | **Sample size** | **Participants** | **Targeted participants with obesity or overweight** | **Timing of outcome assessment** | **Theoretical/ Conceptual framework** | **Social media platform** | **Comparison group** | **Qualitative** | **Outcomes** |
| --- | --- | --- | --- | --- | --- | --- | --- | --- | --- | --- | --- | --- |
| 1 | Brown et al., 2004 | USA | RCT | N=153 (n=102 intervention; n=51 control). Parents: N=69 (n=22 intervention; n=47 for control) | 14-16 years;  F only;  55.6% white, 16.4% multi-ethnic or 'other', 16% Asian, 10.6% Hispanic, 1.4 % African-American | No | Baseline, post-intervention (3 months), follow up (6 months) | Cognitive/behavioral body image interventions; psychoeducational interventions for bulimia of Davis and Olmsted; and healthy weight regulation guidelines suggested by the authors of eating disorder prevention studies | Homegrown website | Wait list control group | Yes | Eating disorder behaviours  **Eating disorder attitudes, behaviours, and symptoms**  Weight concerns scale  **Eating disorder healthy eating, nutrition, exercise, and body image knowledge**  **Parental attitudes and criticism**  Qualitative analysis: parent engagement and satisfaction with intervention |
| 2 | DeBar et al., 2008 | USA | Pre-post analysis of intervention arm of RCT study | N=82 | 14-16 years;  F only;  mostly Caucasian | No | Baseline, during intervention, 12-month follow-up | NR | Homegrown website | No | No. | 24h diet recall – **Calcium intake only**  72 h exercise recall – **High Intensity Interval Training only**  Website usage patterns |
| 3 | Doyle et al., 2008 | USA | RCT | N=80 (n=40 intervention; n=40 control) | 12-18 years; 62.5% F;  50% white, 26.3% Black, 12.5% Hispanic, 11.3% other | Yes | Baseline, post-intervention (4 months), follow-up (8 months) | Cognitive behavioural approaches | Homegrown website | Wait list control provided with informational handouts | Yes | **BMI z-scores for age and gender (CDC)**  **Eating disorder attitudes, behaviours, symptoms**  Frequency of behavioural and cognitive skills (self-monitoring, problem solving, seeking social support, setting goals)  Engagement  Satisfaction  Social support |
| 4 | Jones et al., 2008 | USA | RCT | N=105 (n=52 intervention; n=53 wait-list control) | 69.5% F; 63.8% white, 7.6% Black, 20.9% Latino/Hispanic/Mexican, 7.6% other | Yes | Baseline, 16 weeks, 9 months | Cognitive behavioural approaches and psychoeducation | Homegrown website | Wait list control group | No | **BMI z-score**  **Binge eating behaviour**  **Weight and shape concerns**  Dietary fat and sugar intake  Depressive mood  Program adherence |
| 5 | Whittemore et al., 2013 | USA | Mixed methods evaluation | N=384 | 14-17 years; 61,9% F; 34.6% White non-Hispanic, 21.8% White Hispanic/Latino, 26.3% African-American, 14% Other | Yes | Baseline, 3 months, 6 months | NR | Homegrown website | Same as intervention with the addition of coping skills training | Yes | Reach, adoption, implementation  Satisfaction  Participation data  Qualitative analysis of interviews with teachers |
| 6 | Whittemore et al., 2013 | United States | Cluster RCT | N=384 | 14-17 years; 61.9% F; 34.6% White non-Hispanic, 21.8% White Hispanic/Latino, 26.3% African-American, 14% Other | No | Baseline, 3 months, 6 months | Theory of Interactive Technology/Social Learning Theory/Social Learning Theory | Homegrown website | No coping skills training | No | BMI  Sedentary behaviour*  Physical activity*  Nutrition behaviour*  Self-efficacy*  Satisfaction  Usage |
| 7 | Jones et al., 2014 | USA | Quasi experimental pre- post- test design with control group | N=336 (n=225 healthy weight group; n=111 obesity weight management) | 14 years;  55% Female; 46.7% multiracial /other, 31.0% Caucasian, 16.7% African American, and 5.7% not specified, 43.5% identified as Hispanic or Latino. | No | Baseline, 12 weeks | NR | Homegrown website | One group had content specific to Weight management (overweight or obese participants) while the other focused on Health Habits (healthy weight participants). | No | BMI z-score (CDC)*  Weight and shape concerns  Eating behaviours*  Physical activity* |
| 8 | Kulik et al., 2014 | USA | Pilot RCT | N=36 (n=17 intervention; n=19 enhanced intervention) | 13-17 years;  F only;  58.5% Caucasian; 19.5% Black, African American, 7.3% Hispanic / Latino /Cape Verdean, 4.9% American Indian or Alaska Native; 9.8% other | Yes | Baseline, 4 weeks, 16 weeks | NR | Facebook | Same as intervention, control did not receive online social support component and participated in in-person whole group activities instead. | No | **Support from friends– weight loss (diet and physical activity) specific**  Support from family – weight loss (diet and physical activity) specific  Support from weight loss group peers – general  Group dynamics  Intervention adherence |
| 9 | Lana et al., 2014 | Spain, Mexico | RCT | N=2001 (n=1014 intervention; n=987 control) | 12-15+ years; 54.8% F;  78% Mexican, 22% Spanish | No | Baseline, post-intervention (9 months) | A.S.E. model (Attitude, Social influence and self-Efficacy) and Transtheoretical model | Homegrown website | Yes; NR | No | Total cancer behavioural risk* |
| 10 | Kulik et al., 2015 | USA | Pilot RCT | N=41 (n=18 intervention; n=23 in enhanced intervention) | 13-17 years;  F only;  59% white, 20% Black/African-American, 21% other | Yes | Baseline, 4 weeks, 16 weeks | Peer support approaches | Facebook | Same as intervention group, did not receive access to social media intervention. | No | **Support from friends– weight loss (diet and physical activity) specific**  Support from family – weight loss (diet and physical activity) specific  Support from weight loss group peers – general  Diet  Physical activity  Adherence  BMI and percent overweight |
| 11 | Nawi et al., 2015 | Malaysia | Cluster RCT | N=97 (n=47 intervention; n=50 control) | 16 years; 43.3% F; 78.4% Malay | Yes | Baseline, post intervention (12 weeks) | NR | Homegrown website | Control received printed reading materials from intervention | no | Eating Behaviour Questionnaire  Physical Activity Questionnaire  BMI  Waist circumference  Body fat measurement |
| 12 | Pretlow et al., 2015 | USA | Pilot pre-post- study | N=27 | 16 years;  65% F;  83.7% white | Yes | Baseline, during (40 days, 90 days), post intervention (140 days) | NR | Homegrown application | No | no | **%over BMI**  Program performance and satisfaction  **Control over food**  **Turning to food when stressed**  **Self-esteem** |
| 13 | Sousa et al., 2015 | Portugal | Quasi experimental pre- post- test design with control group | N=71 (n=25 intervention; n=46 control) | 12-18 years; 51% F | Yes | Baseline, during (12 weeks), post intervention (24 weeks) | NR | Homegrown website | Usual care for weight management | No. | BMI z-score (CDC, WHO)  Weekly **physical activity**, screen time  Family support, weight loss motivation, body image  Usability assessment  Adherence to weight control  **Adolescent health-promoting behaviours**  Impact of Weight on Quality of Life |
| 14 | Frerichs et al., 2015 | USA | Pilot pre-post- study | N=74 | NR | No | Baseline, post-intervention (7 weeks) | Social cognitive theory, community mobilization, and social network theory | Facebook | No | No | Confidence to identify healthy foods  Confidence to change eating patterns  Eating behaviours  Qualitative analysis of program inputs and outputs |
| 15 | Chamberland et al., 2017 | Canada | Cluster RCT | N=282 (Intervention 6 clusters n=193 students; Control 4 clusters n=89 students) | 12-14 years; 61% F | No | Baseline, during (daily), post-intervention (9 weeks), follow-up (17 weeks) | Not theory based, however, behaviour change techniques related to self-determination models were used to inform the intervention development e.g. goal settings, providing feedback, identifying barriers and solutions, reinforcement, social support, developing autonomy etc. | Homegrown website | Regular curriculum | Yes | Consumption of fruits and vegetables  Consumption of milk and alternatives  BMI z-score (WHO)  Qualitative focus group data |
| 16 | Park et al., 2017 | USA | Usability testing | N=20 | 13-17 years; 80% F;  70% first generation immigrants;  all Korean American | No | Baseline, during, post-testing | Social cognitive theory (SCT) and the Technology acceptance model (TAM). | Facebook | No | Yes. | Perceived usability  Qualitative analysis of interview and observation data |
| 17 | Chester et al., 2018 | USA | Pilot pre-post | N=118 | 74% F; 32% African American; 61% financially disadvantaged; 74% first-generation to college; 75% rural | No | Baseline, 7 months | 7 R’s: reward, recreation, relationships, research, relevance, rigor, repetition (author developed framework); Social cognitive theory | Facebook | No | No | BMI  Triglycerides  Blood pressure  HDL  Fasting Blood Glucose  Goal setting category |
| 18 | Gonçalves et al., 2018 | Brazil | Pre-post- study | N=69 | 13-19 years; 76.8% F | No | Baseline, 4 months | NR | Facebook | No | No | BMI z-score (WHO)  Body silhouette perception  Body satisfaction |
| 19 | Prout Parks et al., 2018 | USA | Pre-post- feasibility study | N=13 | 14-20 years; 69.2% F;  46.2% African American, 53.8% white | Yes | Baseline, during, 12 weeks | NR | Facebook | No | No | Social media usage  Intervention acceptability  Intervention feasibility  Clinic attrition  Social media engagement |
| 20 | Saez et al., 2018 | France | Process evaluation | N=262 | 13-18 years; 56.5% F | Yes | Baseline, post-intervention (10 months) | The Reader-to-Leader Framework | Facebook | Text message reminders | Yes | Reach  Acceptability questionnaire  Acceptability qualitative interview |
| 21 | Benítez-Andrades et al., 2020 | Spain | Quasi experimental pre- post- test design with control group | N=230 (n=139 intervention; n=91 control) | 11-15 years | No | Baseline, 14 weeks | Social network paradigm | Homegrown application | Educational articles on app website | No | BMI percentile (WHO)  KIDMED  Physical Activity Questionnaire for Adolescents (PAQ-A)  eHealth app usage (entries, responses, likes, healthyStars, interactions) |
| 22 | Januraga et al., 2020 | Indonesia | Qualitative evaluation | N=37 | 16-19 years; F only | No | Post campaign | Technology acceptance model | Instagram, Facebook, YouTube, LINE | No | Yes | Qualitative individual interviews and focus group discussions |
| 23 | Jefrydin et al., 2020 | Malaysia | Quasi experimental pre- post- test design feasibility | N=125 (n=62 intervention; n=63 control) | 13-14 years; 72.8% F; 44% middle income | No | Baseline, 12 weeks | NR | Instagram | No intervention | Yes | Weight  Height  Waist circumference  Nutrition knowledge  Knowledge, attitude, and practice questionnaire on nutrition labelling  Program evaluation survey |
| 24 | Benavides et al., 2021 | Spain | Quasi experimental pre- post- test design with control group | N=301 (n=210 intervention; n=91 control) | 46% F | No | Baseline, 14 weeks | Social network paradigm | Homegrown application | Educational articles on app website | No | BMI percentile (WHO)  KIDMED  Physical Activity Questionnaire for Adolescents (PAQ-A)  Social Network Analysis degrees  eHealth app usage (entries, responses, likes, healthyStars, interactions) |
| 25 | Lin et al., 2021 | Canada | Pre-post formative evaluation | N=301 | 13-17 years; 50.8% F; 67.8% White/European, 32.2% Other; 50.2% <$100 000 CAD income, 39.9% ≥ $100 000 CAD income | No | Baseline; 4.5 months | Social Cognitive Theory, Self-Determination Theory | Homegrown application | No | No | Engagement with app features  Health knowledge  Self-efficacy  Autonomous motivation  Physical activity  Fruit and vegetable and sugar-sweetened beverage intake  Screen time  Sleep |
| 26 | Chae et al., 2022 | South Korea | Non-equivalent randomized controlled trial | N=109 | M=15.7 years (SD=0.61); 54.1% F; 5.5% Low socioeconomic status, 69% Middle socioeconomic status, 25% High socioeconomic status | No | Baseline, post-intervention (3 months) | Transtheoretical model | Wii Fit and unnamed social networking service | Yes | Yes | Weight  Skeletal muscle mass  Body fat %  Body mass index  Waist circumference  High-density lipoprotein cholesterol  Low-density lipoprotein cholesterol  Total cholesterol  Triglyceride  Fasting glucose  Daily step count  Daily sitting time  Programme satisfaction |
| 27 | Felix et al., 2022 | Portugal | Qualitative study | N=16 | 13-18 years; M=15.13 years; 68.8% F | Yes | NA | NR | Facebook | NR | Yes | NA |
| 28 | Rageliene et al., 2022 | Denmark | RCT | N=118 | 9-13 years; M=10.9 years (SD=1.1); 54% F | No | Baseline, post-intervention (3 months) | Mahlke’s user experience model | Homegrown mobile phone application | No intervention | Yes | Availability of healthy food at home  Intake of fruits and vegetables and snacks  Food preference questionnaire  Self-efficacy for healthy eating  Knowledge about healthy eating |
| Note. F= female, M=male; NA=not applicable; BMI=body mass index; RCT=randomized controlled trial; bolded measure outcomes=statistically significant change in the outcomes measured between the two groups compared; * = statistically significant change in outcomes pre-post intervention. | | | | | | | | | | | | |
