# Supplementary material for: Social Media Interventions for Nutrition Education Among Adolescents: Scoping Review
Source: JMIR Pediatr Parent. 2023 Jul 20;6:e36132. doi: 10.2196/36132 (PMC10401194; doi:10.2196/36132)
Supplement: Multimedia Appendix 3 [file pediatrics_v6i1e36132_app3.docx]

**Table S2. Summary of social media intervention characteristics.**

|  | **Author** | **Duration** | **Frequency** | **Social media platform** | **Involved peer leaders or educators or mentors?** | **Involved parents** | **Content** | **Development** | **Additional interventions outside of social media application or website** |
| --- | --- | --- | --- | --- | --- | --- | --- | --- | --- |
| 1 | Brown et al., 2004 | 8 weeks | 1 hour per week during class | Homegrown website | N | Y | Healthy eating; nutrition; exercise; eating disorders; body image; misconceptions about nutrition and exercise needs | NR | None |
| 2 | DeBar et al., 2008 | 2 years | NR | Homegrown website | Y | N | Exercise; nutrition; wellness/stress management; beauty (e.g. skin, hair); fashion | Developed based on adolescents' feedback and preferences indicated during focus group discussions prior to developing website. | None |
| 3 | Doyle et al., 2008 | 16 weeks | 1-2.5 hours per week | Homegrown website | N | Y | Weight loss (e.g. portion sizes, daily activity); body image | NR | None |
| 4 | Jones et al., 2008 | 16 weeks | NR | Homegrown website | N | Y | Binge eating; maintaining weight; healthy eating; physical activity; reducing sedentary activities | NR | Weekly letters, face-to-face meetings with mentor in second cohort due to low participation |
| 5 | Whittemore et al., 2013; Whittemore et al., 2013 | 6 months | 8-12 lessons | Homegrown website | N | N | Healthy eating; physical activity  Nutrition; physical activity; metabolism; portion control | NR | None |
| 6 | Jones et al., 2014 | 12 weeks | 12 sessions | Homegrown website | N | Y | Healthy eating; binge eating; body image | User-centred design process using prototypes, focus groups, and usability testing. | Newsletters, school curriculum, in-person meetings, |
| 7 | Kulik et al., 2015; Kulik et al., 2014 | 16 weeks | Weekly, then biweekly, then monthly | Facebook | Y | Y | Nutrition; physical activity; behaviour skills; cognitive approaches  Diet and exercise for weight loss | NR | Weekly face-to-face group sessions, weight management clinic |
| 8 | Lana et al., 2014 | 9 months (entire academic year) | NR | Homegrown website | N | N | Healthy diet; preventing cancer risk behaviours | NR | Text messages reminders |
| 9 | Nawi et al., 2015 | 12 weeks | NR | Homegrown website | N | Y | Healthy living; weight loss | The website content were developed by a panel expert consisting of a psychologist, public health specialist, two dietitians, and a nurse. | Weight management program at tertiary clinic |
| 10 | Pretlow et al., 2015 | 20-week program | NR | Homegrown mobile phone application | N | N | Identifying problem foods; snacking; excessive food | NR | Weekly phone meetings with mentor; regular face-to-face group meetings |
| 11 | Sousa et al., 2015 | 24 weeks | 1 module every 2 weeks | Homegrown website | N | Y | Weight management skills; health promoting skills | NR | None |
| 12 | Frerichs et al., 2015 | 7 week period | NR | Facebook | Y | N | Food labelling; healthy food choice | Youth led and designed campaign and intervention. | Food labelling system implemented in school |
| 13 | Chamberland et al., 2017 | 6 weeks | NR | Homegrown website | N | N | Improving diet and nutrition | NR | None |
| 14 | Park et al., 2017 | NR | NR | Facebook | N | N | Obesity; healthy eating; physical activity | The program content was developed by a team of experts in adolescent health and obesity, healthy eating and physical activity, and the Korean adolescents, involving literature review, needs assessment and iterative refinements based on feedback from Korean adolescents on the team. | None |
| 15 | Chester et al., 2018 | 7 months | Unrestricted access to Facebook group with weekly prompts and tasks to complete | Facebook | Y | N | Self-regulation skills, diet and exercise, goal setting, coping mechanisms for stress and emotional difficulties | NR | None |
| 16 | Gonçalves et al., 2018 | 4 months | Once every 2 days or 30 mins daily | Facebook | N | N | NR | Designed by teacher/educator. | None |
| 17 | Prout Parks et al., 2018 | 12 weeks | Weekly, 3 times weekly | Facebook | N | N | Nutrition, physical activity, behavioral modification topics, cooking, and exercise | NR | Weight management program at tertiary clinic; 2 in-person meetings with group members |
| 18 | Saez et al., 2018 | Academic year | 2 challenges per week | Facebook | Y | N | Healthy eating; physical activity | NR | Text messages reminders |
| 19 | Benítez-Andrades et al., 2020; Benavides et al., 2021 | 14 weeks | Unrestricted access to application | Homegrown mobile phone application | N | Y – suggestions to involve parents in activities. | Personalized healthy eating and physical exercise tips | NR | Virtual rewards for healthy goal setting; creating healthy events |
| 20 | Januraga et al., 2020 | NR | NR | Instagram | N | N | Activities, articles, recipes, and photos related to healthy eating | NR | Website with information related to Instagram content and BMI calculator |
| 21 | Jefrydin et al., 2020 | 12 weeks | Unrestricted access to educational content, education posts every week and regular Instagram stories to promote participation | Instagram | N | N | Health eating, nutrition | Content designed based on a needs assessment. | Weekly quizzes testing participants knowledge |
| 22 | Lin et al., 2021 | 4.5 months | Unrestricted access to the application | Homegrown mobile phone application | N | Y | Improving dietary quality, increasing physical activity, reducing screen time, improving sleep | Developed from first- and second-generation versions of a modular online program addressing childhood obesity. Refined using qualitative feedback from studies of the previous generations. | Application allows adolescents to work through goals and modules they choose. Check-ins, quizzes, activities, virtual world currencies, and interactive stories are used to deliver the topic modules. |
| 23 | Chae et al., 2022 | 12 weeks | Weekly interactive small group discussions on social networking service | Wii Fit and unspecified social networking service | N | N | Healthy behaviours, especially physical activity and healthy dietary behaviours | Developed based on qualitative needs assessment with adolescents and school health professionals, and input during development process from school health professionals | 30 minutes of exergaming 3 times weekly  24-hour diet diary twice a week  Weekly text message nutrition counselling  50-minute classroom lesson once |
| 24 | Felix et al., 2022 | NR | Monthly topics delivered over private Facebook group with daily posts | Facebook | N | N | Physical activity, healthy eating, management of problematic eating behaviours, self-esteem | NR | Weekly self-monitoring questionnaire with automatic personalized feedback  Option to schedule monthly online chats with a psychologist |
| 25 | Rageliene et al., 2022 | 3 months | Unrestricted access to the application | Homegrown mobile phone application | N | N | Healthy eating | Application was developed and then refined using a pre-study with a small sample of youth | Food diary  Mobile phone application games |
