# Supplementary material for: Social Media Interventions for Nutrition Education Among Adolescents: Scoping Review
Source: JMIR Pediatr Parent. 2023 Jul 20;6:e36132. doi: 10.2196/36132 (PMC10401194; doi:10.2196/36132)
Supplement: Multimedia Appendix 4 [file pediatrics_v6i1e36132_app4.docx]

**Table S3. Feasibility outcomes.**

|  | **Author** | **Recruitment rate** | **Dropout rate / attrition** | **Engagement** | **Cost** |
| --- | --- | --- | --- | --- | --- |
| 1 | Brown et al., 2004 | NR | NR | 11/22 parent participants logged onto website, 8/11 read through most of the intervention content.  Adolescent participant engagement NR. | NR |
| 2 | DeBar et al., 2008 | NR | NR | 23.2 website visits on average over 2 years (SD 38.6, range 0-233).  112.1 minutes of website use on average over 2 years (SD 138.6, range 0-623).  4.4 minutes of self-reported website use per week (SD 4.9, range 0-22.5). | Picture caption contest where participants could win monthly prizes, take pop-up quizzes, and ‘count up’ and ‘cash in’ incentive points (redeemable with local merchants) they earned for project participation. |
| 3 | Doyle et al., 2008 | 80 participants over 7 months | 18% for both arms | Participants read 29.9% of the intervention material on average (range 0-90.7%, SD 27.3).  14 participants (35%) viewed less than 10% of the intervention material. | Several $20 gift cards as prizes for adherence to intervention |
| 4 | Jones et al., 2008 | NR | 17% | Mean 20.2 website pages viewed out of 104 (SD 28.2, range 0-94)  Mean 4.5 weeks of content accessed out of 16 weeks (SD 5.2, range 0-16)  Mean 5.2 discussion group postings (SD 8.6, range 0-34)  Mean 21.4 food journal entries (SD 30.7, range 0-107)  Mean 8.4 physical activity journal entries (SD 23.4, range 0-157)  Mean 2.8 weight journal entries (SD 4.3, range 0-16)  Mean 3.0 personal journal entries (SD 3.0, range 0-23)  27% of participants (14/52) used some intervention component for 8 weeks.  42% of participants (22/52) used intervention for 1 to 7 weeks.  31% of participants (16/52) never used intervention. | NR |
| 5 | Whittemore et al., 2013; Whittemore et al., 2013 | 384 participants over 4 months | 5% | NR | Study participants received a gift card for completion of data collection ($25.00 at baseline; $30.00 at follow-up) |
| 6 | Jones et al., 2014 | Students provided with forms at the beginning of school week and had to return by end of week to be enrolled in the study. 336 participants over 1 week. | NR | NR | NR |
| 7 | Kulik et al., 2014; Kulik et al., 2015 | NR | 11% | In person session attendance on average 7.72 (SD .74) out of 8 sessions.  On average 6.45 (SD 2.9) contacts out of 15 with group leader.  None of the participants completed all 13 Study Buddy chats.  4/19 participants did not do any of the 13 Study Buddy chats.  6/19 participants completed half of the 13 Study Buddy chats.  4/19 participants completed half of the 45 possible check-ins with other group members. | NR |
| 8 | Lana et al., 2014 | NR | 58.5% intervention arms  68% control arm | NR | NR |
| 9 | Nawi et al., 2015 | NR | No dropouts | NR | NR |
| 10 | Pretlow et al., 2015 | NR | 37% | 8/35 participants did not comply with intervention and were removed. | Each recruited participant was supplied with an iPhone 4S, a wireless Bluetooth body weight scale interfaced to the app, and a digital food scale. Each participant also compensated up to $200 maximum according to the number of outcome measures completed. |
| 11 | Sousa et al., 2015 | NR | 48% | 1/28 parent participants accessed the platform.  10.68 visits on average to platform (SD 18.92) over 24 weeks and recorded 3.00 data points on average (SD 4.85) over 24 weeks.  Participants analyzed 7.87 resources on average (SD 9.25) and read an average of 31.77 messages from the forum (SD 47.56) over 24 weeks and completed 13.66% of activities on average (SD 47.56). | NR |
| 12 | Frerichs et al., 2015 | NR | NR | Increase in Facebook page likes from 36 to 90. Weekly reach increased from 61 pre-intervention to 322 post-intervention. | NR |
| 13 | Chamberland et al., 2017 | NR | NR | NR | NR |
| 14 | Park et al., 2017 | 20 participants over 3 months | NA | NA | $25 gift certificate to participants for testing session |
| 15 | Chester et al., 2018 | NR | 51.7% | 57/118 participants returned for follow-up and used social media 44 times.  61/118 participants did not return for follow-up and used social media 14 times. | Students and mentors could earn up to $50 USD for their participation. |
| 16 | Gonçalves et al., 2018 | NR | NR | Mean 31 views per admin post.  55% of adolescents participated in at least one activity.  383 likes and 55 comments throughout the intervention. | NR |
| 17 | Prout Parks et al., 2018 | NR | No dropouts | Mean total likes over 12 weeks 43.1 (SD 23.5, range 9-82)  Mean total comments over 12 weeks 12.9 (SD 3.43, range 3-18)  Mean total engagement (likes and comments) over 12 weeks 56 (SD 24.3, range 14-85)  Mean weekly engagement 8.6 (SD 3.6, range 14-85) | $25 monthly incentive to assist with mobile data cost for active participants |
| 18 | Saez et al., 2018 | NR | 22% | 64.1% (168/262) participated in at least 1 in-person session.  8% (21/262) participated in the Facebook group. | NR |
| 19 | Benítez-Andrades et al., 2020; Benavides et al., 2021 | 511 invited, 307 enrolled, | 2% | Total 7696 application sessions over 14 weeks, average 58.75 per person, 80.17 per day.  Total 1127 friend requests over 14 weeks, average 8.60 per person, 11.74 per day.  Total 181 accepted friend requests over 14 weeks, average 1.38 per person, 1.89 per day.  Total 222 rejected friend requests over 14 weeks, average 1.70 per person, 2.31 per day.  Total 3722 posts over 14 weeks, average 28.41 per person, 38.77 per day.  Total 4727 likes over 14 weeks, average 36.08 per person, 49.24 per day.  Total 107 events over 14 weeks, average 0.82 per person, 1.12 per day.  Total 11215 reward points over 14 weeks, average 85.61 per person, 116.82 per day. | NR |
| 20 | Januraga et al., 2020 | NR | NR | NR | NR |
| 21 | Jefrydin et al., 2020 | NR | NR | NR | NR |
| 22 | Lin et al., 2021 | 1644 parent-teen dyads approached; 1418 dyads screened; 873 eligible; 632 expressed interest in study; 426 consented and assented; 301 enrolled | NR | 31.9% did not use most app features;  25.3% minimally used all app features;  23.9% high use of app features;  18.9% very high use of app features  Average minutes of app use dropped substantially week 2 onwards. Percentage of teens engaged with app declined at the same rate across 20 weeks for the two high usage groups. Percentage of teens engaged with app declined substantially from week 3 onwards for the two low usage groups. | NR |
| 23 | Chae et al., 2022 | 127 enrolled; 109 included in final analysis | NR | NR | Participants provided with Wii Fit. |
| 24 | Felix et al., 2022 | NR | NR | NR | NR |
| 25 | Rageliene et al., 2022 | 300 enrolled | 68% intervention; 55.6% control | Participants reported using the application for 5-15 minutes per session. | For qualitative study participants received cinema ticket. For baseline data collection in efficacy evaluation, participants had a 10% chance of winning a cinema ticket. |
| Note: NA – not applicable; NR – not reported | | | | | |
