# Supplementary material for: Social Media Interventions for Nutrition Education Among Adolescents: Scoping Review
Source: JMIR Pediatr Parent. 2023 Jul 20;6:e36132. doi: 10.2196/36132 (PMC10401194; doi:10.2196/36132)
Supplement: Multimedia Appendix 5 [file pediatrics_v6i1e36132_app5.docx]

**Table S4. Factors influencing implementation and success of interventions.**

| Role of families and communities |
| --- |
| - Intervention content may need to address strategies to manage interpersonal barriers such as peer pressure, lack of parental support for healthy eating habits, lack of control over food at home and when going out with friends, and tensions with valuing home-cooked cultural foods and healthier food choices. - A high level of participation and engagement with the intervention among parents may not be necessary to elicit positive impacts among participants. Adolescents may involve their families through sharing according to their comfort levels. Parental involvement in a program using social media is also subject to families’ digital literacy. Parental involvement in the intervention can also detract from the independence adolescents may be attempting to achieve in this period of development. - Including family members in the intervention design can ensure family wide behaviour change. Programs may elicit family wide and peer network wide behaviour change when participants share what they learn with friends and family and model healthy behaviours. - Designing interventions in concert with community organizations, schools, and health systems ensures it is seamless with existing work in communities. Community partners may aid in planning and delivery of intervention, can provide feedback on improvements to the intervention, and ensure sustainability of the intervention. - Mothers may support behaviour change more frequently than fathers due to gender norms and changes in behaviour may be associated with their involvement. |
| Tailoring for target population |
| - Social media platform should be selected based on the target group, differences may exist between males and females; different socioeconomic status levels; age groups; and countries. - Adolescent populations prefer content that is presented concisely and simply, in actionable terms, and offers practical applications, and programs that offer frequent opportunities to interact with peers. Content should demonstrate how healthy food can be made to taste good and recipes that involve minimal ingredients, low-cost ingredients, and are simple to prepare. - Interventions should be relevant to adolescents’ cultural backgrounds and socioeconomic status to improve utility of the program to them. - Content should be tailored to address low self-efficacy and include positive message framing. Healthy eating should be framed as part of improving health and maintaining a healthy lifestyle versus a focus on appearance related outcomes of healthy eating. Content should be reviewed to avoid stigmatizing males, females, and cultural foods and practices. - Interventions for mixed weight populations should have an adequate amount of health promoting features for both populations that are overweight or obese and healthy weight populations. - Including adolescents’ input and/or as design partners at all stages of the research is an effective way of ensuring relevance of the intervention to youth. - In some certain countries, a barrier to participation in programs is the high focus on adolescent age children’s academic achievement, reducing time available for non-academic activities. |
| ***Engagement*** |
| - Participants are less motivated to engage with interventions when they do not involve existing peer networks. Interventions should incorporate more elements of peer support to learn from one another. Peers’ engagement also facilitates participants’ engagement in the intervention. - Interventions should be delivered on social media platforms already used by youth and strategies such as text, WhatsApp or Instagram notifications and messages can prompt engagement with interventions on these existing platforms. - In-person meetings as adjunct to the online intervention may improve engagement along with peer leaders, educators, and mentors who offer a point of contact and build rapport with participants. - An abundance of guidance in the intervention can facilitate engagement. Joining and participating in the intervention should avoid complicated processes. - More dynamic and responsive social media environments are needed to maintain adolescents’ engagement. Intervention content should avoid requirements of completing long lessons and modules. - High levels of adherence and programs longer than 12-16 weeks may not be needed to elicit improvements in health outcomes, programs may need to front-load curricula to maximize effectiveness. - Participants engage with content by liking content and sharing with online and offline friends and family. Participants may gain benefit from the program through more passive engagement “lurking”. - During a pandemic, interest can wax, and wane and effectiveness can improve due to quarantine and isolation. - Improvements in dietary behaviours may be elicited even with low or passive engagement with the intervention. Engagement may also occur outside of the digital environment. |
| ***Technical and logistical issues*** |
| - Use platforms that participants already use and move to new platforms and features as social media trends change. - Test and understand features and functionality of social media to address them ahead of the intervention. - Counsel participants on privacy and safety online and create a privacy and safety contingency plan. - Ensure participants have access to devices and internet. - Some platforms may be chosen according to technical requirements – participants may want a platform that uses less data as they rely on parents for Internet access. - Programs may offer cross-platform integration, one platform may be better suited for certain types of content. |
| Peer support |
| - Nutrition education delivered via social media must make use of peer support to facilitate sustainable changes in health behaviours. Peers may support one another during behaviour change and facilitate learning new practical skills and contribute to the positive impact of the intervention. However, peer support should be drawn from existing social networks. - A number of mechanisms of influence may explain the role of peer support in health behaviour change e.g., contagion, acceptance of group norms, weak ties, peer modeling, imitation etc. Thus, there is uncertainty surrounding how interventions should be designed to facilitate health behaviour change through social networks. |
